# The gut microbiome in Alzheimer’s disease: what we know and what remains to be explored

**DOI:** 10.1186/s13024-023-00595-7

**Published:** 2023-02-01

**Authors:** Sidhanth Chandra, Sangram S. Sisodia, Robert J. Vassar

**Affiliations:** 1 Ken and Ruth Davee Department of Neurology, Northwestern University Feinberg School of Medicine, Chicago, IL 60611 USA; 2 Medical Scientist Training Program, Northwestern University Feinberg School of Medicine, Chicago, IL 60611 USA; 3 Department of Neurobiology, University of Chicago, Chicago, IL 60637 USA

**Keywords:** Gut microbiome, Amyloid, Tau, Neuroinflammation, Peripheral immunity, Human, Mouse, Therapeutics, Diet, Sleep, Exercise

## Abstract

Alzheimer’s disease (AD), the most common cause of dementia, results in a sustained decline in cognition. There are currently few effective disease modifying therapies for AD, but insights into the mechanisms that mediate the onset and progression of disease may lead to new, effective therapeutic strategies. Amyloid beta oligomers and plaques, tau aggregates, and neuroinflammation play a critical role in neurodegeneration and impact clinical AD progression. The upstream modulators of these pathological features have not been fully clarified, but recent evidence indicates that the gut microbiome (GMB) may have an influence on these features and therefore may influence AD progression in human patients. In this review, we summarize studies that have identified alterations in the GMB that correlate with pathophysiology in AD patients and AD mouse models. Additionally, we discuss findings with GMB manipulations in AD models and potential GMB-targeted therapeutics for AD. Lastly, we discuss diet, sleep, and exercise as potential modifiers of the relationship between the GMB and AD and conclude with future directions and recommendations for further studies of this topic.

## Background

Alzheimer’s disease (AD) is a neurodegenerative disorder that is the most common cause of dementia and currently has few clinically efficacious disease modifying therapies [1]. In order to develop disease modifying therapies, it is imperative to better understand the mechanisms of disease initiation and progression. The pathological hallmarks of AD include senile plaques composed of amyloid beta (Aβ) peptides, neurofibrillary tangles composed of hyperphosphophorylated forms of the microtubule-associated tau protein, and neuroinflammation that lead to neurodegeneration [1]. Neuroinflammation has been linked to the development and progression of these disease pathologies. The gut microbiome (GMB) is comprised of trillions of bacteria, archaea, protozoa, viruses, and fungi and has been shown to potentially regulate neuroinflammation in a variety of neurological conditions, including Multiple Sclerosis [2], Parkinson’s disease [3, 4], and AD [5–8].

**Fig. 1:**
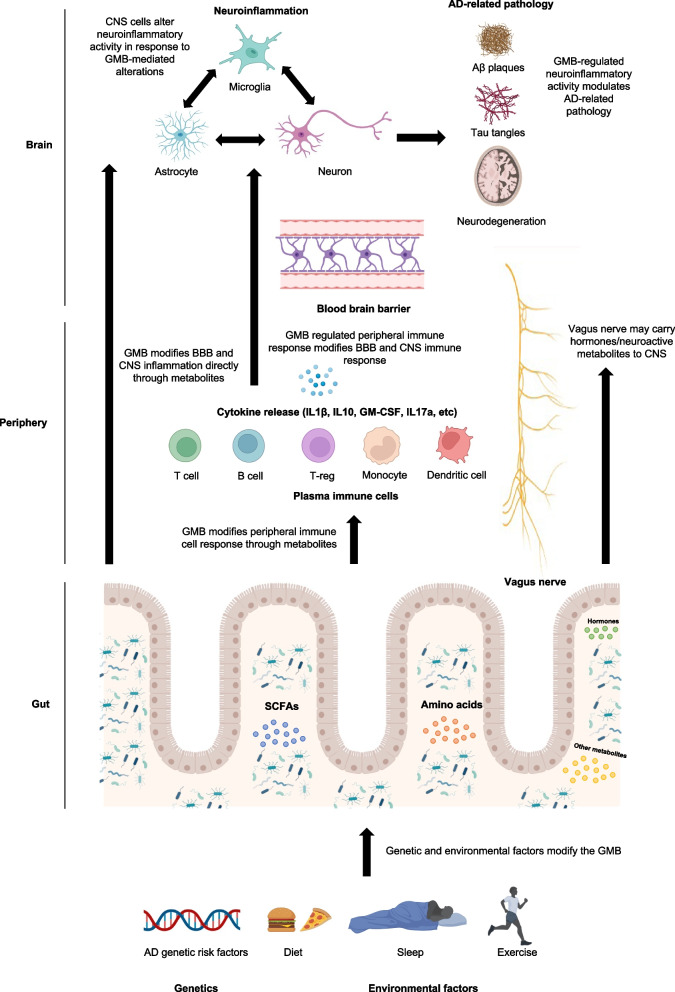
Theory of GMB involvement in AD. Environmental factors, such as diet, sleep, and exercise and the development of AD due to genetics contribute to an inflammatory environment in the gut microbiota, which leads to changes in composition and diversity over time. Changes in the gut microbiota influence gut microbiome-derived metabolites and peripheral immunity by altering peripheral immune cell gene expression and cytokine release. Changes in peripheral immunity, possibly gut-derived metabolites directly, and vagus nerve trafficked gut-derived hormones can then alter phenotype of the blood brain barrier and central nervous system cell types (microglia, astrocytes, neurons), which can then modulate amyloidosis, tauopathy, and neurodegeneration and contribute to disease pathogenesis

GMB-mediated regulation of neuroinflammation may happen through direct or indirect mechanisms (Fig. 1). Changes in the GMB can alter microbial-derived metabolites and peripheral immunity, which could then potentially alter CNS immune response in the context of neurological disease (Fig. 1) [9]. While recent studies suggest AD patients have an altered GMB compared with those without AD [10, 11], and manipulations to the GMB in mouse models of AD can alter pathology and neuroinflammation [5–8, 12–14], the precise mechanisms by which the GMB influences AD remain to be elucidated. In this review, we summarize studies in AD patients and mouse models of Aβ amyloidosis which implicate the GMB in AD pathogenesis (Fig. 1). Furthermore, we highlight potential mediators of the relationship between the GMB and AD that need further study. We also discuss microbiome-mediated therapeutic strategies for AD that may modify disease progression. Finally, we discuss the future of GMB research in the AD field and important tools that will be needed to make progress toward the goal clarifying the roles of the GMB in AD.

### Human evidence of GMB alterations in AD

Two initial studies in 2017 showed that amyloid-positive individuals/AD patients have an altered GMB composition compared to individuals without amyloid/AD. Cattaneo and colleagues (N = 83 total, 40 amyloid positive with cognitive impairment, 33 amyloid negative with cognitive impairment, 10 amyloid negative without cognitive impairment) measured plasma levels of RNAs encoding selected cytokines and stool abundance of particular GMB taxa (*Escherichia/Shigella, Pseudomonas aeruginosa, Eubacterium rectale, Eubacterium hallii, Faecalibacterium prausnitzii, Bacteroides fragilis*) using qPCR approaches (Table 1) [10]. These investigations revealed an increase in mRNA encoding pro-inflammatory cytokines IL6, CXCL2, NLRP3, and IL1β and a decrease in mRNA encoding anti-inflammatory cytokine IL-10 in amyloid-positive patients compared with amyloid-negative individuals. A positive correlation was observed between the pro-inflammatory cytokines and *Escherichia/Shigella* (which has been previously associated as a pro-inflammatory taxon) and a negative correlation with *Eubacterium rectale* (which has been previously associated as an anti-inflammatory taxon). Furthermore, Vogt and colleagues (*N* = 50 total, 25 AD, 25 healthy controls), performed unbiased 16 s ribosomal RNA amplicon sequencing on DNA isolated from fecal matter from AD patients with dementia and healthy age and sex-matched control subjects (HC) (Table 1) [11]. Here, a decrease in GMB bacterial diversity was observed in AD patients, as well as a decrease in *Firmicutes* and *Bifidobacterium* and increased levels of *Bacteroidetes* compared with HC. Since these initial studies, two other studies from groups in China also found alterations in GMB composition between AD patients and HC [15, 16]. Zhuang et al. (*N* = 86, 43 AD, 43 healthy controls) found a decrease in *Bacteroidetes* and an increase in *Actinobacteria* in AD patients compared with HCs (Table 1) [15]. Liu et al. (*N* = 97 total, 33 AD, 32 MCI, 32 healthy controls) found a reduction in *Firmicutes* and an increase in *Proteobacteria* in AD patients compared with controls (Table 1) [16]. Taken together, the results from these studies suggest that GMB composition is altered in AD patients and that GMB changes may have an influence on the progression of AD. However, these studies are entirely correlative and results from human AD microbiome-mediated therapeutic clinical trials will be needed to assess whether the alterations in the GMB directly influence AD pathogenesis. Additionally, there seems to be little consensus between the particular bacterial phyla that are altered in AD patients in these studies. Moreover, the patients and controls in the published studies are quite small (< 50 participants/group) and there are several confounding factors that might influence patient GMB composition including geographical location, diet, and environmental exposures. To resolve these confounding issues, we propose that it will be critical to carry out a large, international study that assesses GMB composition between age and sex-matched AD patients (preferably divided by MCI/AD stage) and HCs. It will also be important that stool collection protocols are standardized, and stool consistency is accounted for as this can alter GMB composition results [17].

**Table 1:** Bacteria altered in human AD patients compared to controls

Study	Bacteria altered	Sample size	Location	Age-matched (y/n)	Sex-matched (y/n)	Reference number
Cattaneo et al. (2017)	Amyloid positive individuals had lower Eubacterium rectale and higher Escherichia/Shigella compared with both healthy controls and amyloid negative groups.	*N* = 83 total, 40 amyloid positive with cognitive impairment, 33 amyloid negative with cognitive impairment, 10 amyloid negative without cognitive impairment	Eastern Lombardy, Italy	y	y	[10]
Vogt et al. (2017)	AD patients had a decrease in Firmicutes and Bifidobacterium and increased levels of Bacteroidetes compared with healthy controls.	*N* = 50 total, 25 AD, 25 healthy controls	Wisconsin, USA	y	y	[11]
Zhuang et al. (2018)	AD patients had a decrease in Bacteroidetes and an increase in Actinobacteria in AD patients compared with healthy controls.	*N* = 86, 43 AD, 43 healthy controls	Chongqing, China	y	y	[15]
Liu et al. (2019)	AD patients had a reduction in Firmicutes and an increase in Proteobacteria compared with healthy controls.	*N* = 97 total, 33 AD, 32 MCI, 32 healthy controls	Hangzhou, China	y	y	[16]

### Evidence of GMB alterations in AD mouse models and effects of GMB manipulations on pathology

#### Changes in AD mouse GMB

Consistent with alterations of GMB composition observed in AD patients, differences in the GMB have also been observed in AD mouse models, including 5XFAD [18, 19], APPSwe/PSEN1dE9 (APP/PS1) [20–23], and APPSwe/PSEN1L166P (APPPS1-21) [14] models compared with wildtype mice (Table 2). Brandscheid et al. (2017) reported an increase in *Firmicutes* and a decrease in *Bacteroidetes* phyla at 9 weeks in 5XFAD mice compared to wildtype mice (Table 2) [18]. On the contrary, Chen et al. (2020) reported a decrease in *Firmicutes* and an increase in *Bacteroidetes* phyla at 3 months in 5XFAD mice compared with wildtype mice (Table 2). At 6 months, there was a marked increase in *Bacteroidetes*, *Proteobacteria*, and *Deferribacteres* [19]. Additionally, a decrease in alpha diversity was observed [19]. Shen et al. (2017) profiled the GMB in 3, 6, and 8-month-old APP/PS1 mice. Age-dependent changes in the GMB that roughly correlated with amyloid pathology were observed: an increase in *Odoribacter* and *Helicobacter* genera and decreases in *Prevotella* species (Table 2). Furthermore, an age-dependent decrease in GMB diversity was also observed [20]. Chen et al. (2020) assessed the GMB profiles in 1, 2, 3, 6, and 9-month-old APP/PS1 mice (Table 2). GMB changes in APP/PS1 mice were observed as early as 1 month and increased over time. Here, significant increases in *Escherichia-Shigella*, *Desulfovibrio*, *Akkermansia*, and *Blautia* in APP/PS1 mice were observed over time [21]. In Zhang et al. (2017), *Verrucomicrobia* and *Proteobacteria* were found to be increased in 8–12-month-old APP/PS1 mice (Table 2). However, *Ruminococcus* and *Butyricicoccus* were significantly decreased in 8–12-month-old APP/PS1 mice compared with wildtype mice. Interestingly, several short chain fatty acids (SCFAs) were decreased in the feces and brain of APP/PS1 mice compared with wildtype mice [22]. Cuervo-Zanata et al. (2021) reported sex-specific changes in APP/PS1 mice compared with wildtype mice. Surprisingly, male APP/PS1 mice had larger alteration in their GMB profiles compared with females [23]. Harach et al. (2017) reported significant increases in *Bacteroidetes* and *Tenericutes* and a decrease in *Firmicutes*, *Verrucomicrobia*, and *Proteobacteria* at 8 months in APPPS1-21 mice compared with wildtype mice [14]. Overall, these studies indicate that there are likely age and sex-dependent changes in GMB composition and diversity between mouse models of Aβ amyloidosis and their wildtype counterparts. While of interest, there is an inherent caveat in trying to ascertain the importance of specific bacterial species and changes in composition because the animal models and wild-type counterparts that are housed in different mouse facilities, given different diets, and possessing different genetic backgrounds will most certainly have differing GBMs in respective mouse colonies.

**Table 2:** Bacteria altered in AD mouse models compared to controls

Study	Bacteria altered	Sample size	Model	Age	Sexes	Reference number
Brandscheid et al. (2017)	Observed an increase in Firmicutes and a decrease in Bacteroidetes phyla at 9 weeks in 5XFAD mice compared to wildtype mice. There was no change in GMB at 6 or 18 weeks in 5xFAD compared to wildtype.	6 per group	5XFAD	6 weeks, 9 weeks, 18 weeks	Male	[18]
Chen et al. (2020)	Observed a decrease in Firmicutes and an increase in Bacteroidetes phyla at 3 months in 5XFAD mice compared with wildtype mice. At 6 months, there was a marked increase in Bacteroidetes, Proteobacteria, and Deferribacteres.	5 per group	5XFAD	3, 6 months	Male	[19]
Shen et al. (2017)	An increase in Odoribacter and Helicobacter genera and decreases in Prevotella species was observed.	6 per group	APP/PS1	3, 6, 8 months	Male	[20]
Chen et al. (2020)	Increases in Escherichia-Shigella, Desulfovibrio, Akkermansia, and Blautia in APP/PS1 mice were observed over time starting at 1 month of age.	2 per group	APP/PS1	1, 2, 3, 6, 9 months	Male	[21]
Zhang et al. (2017)	Verrucomicrobia and Proteobacteria were found to be increased and Ruminococcus and Butyricicoccus were significantly decreased in 8–12-month-old APP/PS1 mice.	6 per group	APP/PS1	8–12 months	Male	[22]
Cuervo-Zanatta et al. (2021)	Sex-specific changes in APP/PS1 mice compared with wildtype mice were observed. At phylum level, TG F had more Bacteriodetes than TG M. At genus level, there were sex-specific changes in Klebsiella, Lactobacillus, Lactococcus, and SMB53.	6–9/group	APP/PS1	6 months	Male and Female	[23]
Harach et al. (2017)	Reported significant increases in Bacteroidetes and Tenericutes and a decrease in Firmicutes, Verrucomicrobia, and Proteobacteria at 8 months in APPPS1-21 mice compared with wildtype mice.	6–8/group	APPPS1-21	1, 3.5, 8 months	Male and Female	[14]

#### Amyloidosis

In addition to assessment of GMB composition between AD mouse models and wildtype mice, several studies have altered the GMB in AD mouse models, primarily by using antibiotics (abx) or housing mice in germ-free environments wherein the mice are devoid of microbes and thus do not develop a GMB. Abx has been shown in several AD mouse models to alter GMB composition, including the APP/PS1 [5, 6], APPPS1-21 [7, 8, 24], 5XFAD [12, 25, 26], and APP^NL−G−F^[27] models. For example, Minter et al. (2016) [5] exposed mice between P14-P21 with a high dose of broad spectrum abx cocktail and observed a change in GBM composition with a marked increase in *Akkermansia* and *Lachnospiracea* at 6 months of age compared with vehicle-treated controls. Importantly, Abx-mediated alteration in GMB composition requires an abx cocktail, as individual abx are not effective at altering either GMB composition or amyloidosis [24]. Moreover, the abx used in the cocktail do not cross the blood brain barrier, thus indicating that the effects on Aβ amyloidosis are mediated by the GMB rather than by direct effects in the brain [5–8, 12, 19, 24]. Additionally, three studies have derived APPPS1-21 [13, 14] and 5XFAD mice [12] in germ-free settings, where the mice are devoid of GMB, and compared them to conventionally raised animals that have an intact GMB. GMB perturbation studies in AD models have found that manipulation of the GMB with abx results in a reduction of Aβ deposition regardless of the model [5–8, 12, 19, 24, 25, 27]. This reduction in Aβ deposition (and insoluble Aβ levels) has been validated using quantitative immunohistochemistry, western blotting, enzyme-linked immunosorbent assay (ELISA) and meso scale discovery (MSD) assays. Importantly, this reduction in amyloid deposition is sex-specific and only occurs in males in the APPPS1-21 and APP/PS1 mouse models [7, 8]. There are several potential reasons for these sex-specific outcomes that need further investigation. For example, differences in GMB-hormone interactions [28, 29] as well as differences in immune response between sexes [30] might be responsible. Importantly, it has been reported that administration of fecal matter transplants (FMT) from non-abx treated amyloid model mice into abx-treated amyloid model mice restores their GMB and Aβ pathology, showing that GMB perturbations are indeed causing alterations in amyloidosis as opposed to non-specific abx effects on amyloidosis [7, 8]. The abx-mediated reduction in amyloid is consistent with changes observed in germ-free mice [12–14]. Germ-free mice also have a strong reduction in amyloid beta pathology, but surprisingly, this effect is not sex-specific [12–14]. The mechanism by which abx treatment decreases Aβ pathology in males but not females as opposed to equal reduction of amyloid in GF mice is not known. However, recent evidence indicates that the immune system and microglia in GF mice are in a very immature state and that differences in the biology of microglia in males and females raised in SPF vs GF conditions would be very different. In this regard, Thion et al. (2018) [31] have reported that the microbiota influences in prenatal and adult microglia in a sex-specific manner. Supporting these findings, Gunekaya et al. (2018) [32] reported that there are transcriptional and translational differences in microglia in brains of male and female animals. One important aspect of abx-treated animals versus GF animals is that in contrast to GF animals that lack GBM, abx does not necessarily lead to GBM depletion but rather to a change in bacterial composition and diversity. Importantly, when GF mice are recolonized with microbiota, amyloid pathology is restored [13, 14]. The mechanism whereby the GMB influences amyloidosis appears to be independent of changes in amyloid beta processing machinery, as there seems to be minimal whole brain changes in amyloid precursor protein (APP) or beta-secretase 1 (BACE1) between abx-treated mice or GF mice and conventionally raised non-treated mice [5, 6, 14]. Notably, Harach et al. (2017) reported that the levels of Aβ degrading enzymes, such as neprilysin and insulin degrading enzymes are increased in GF APPPS1-21 mice compared to conventionally housed APPPS1-21 mice [14], and hence, it is plausible that the increase in Aβ degrading enzymes could be responsible for the GF and abx mediated reduction in amyloid pathology.

#### Neuroinflammation

It has been consistently observed that abx or GF mediated GMB depletion or absence, respectively, in amyloidosis models results in changes in microglial inflammatory state [5–7, 12–14, 24]. Several studies have shown that at the time of cull, microglia appear to become less pro-inflammatory and more phagocytic as determined by RNAseq and microglial morphological analysis in the context of abx-mediated compositional changes in the GMB [6, 7, 24]. Specifically, APP/PS1 mice treated with abx have reduced plaque-associated microglia and altered microglial morphology in which microglia have increased process length and number, consistent with a more homeostatic state [6, 7, 24]. APPPS1-21 mice treated with antibiotics have a similar microglial phenotype and as abx-treated APP/PS1 mice, and bulk RNAseq also revealed that these microglia have a reduction in microglial cell activation by gene ontology analysis [7, 8]. Importantly, microglial changes are restored when abx-treated mice are given FMT from non-treated mice [7, 8]. Taken together, these results suggest that microglia in the context of abx may lose deleterious pro-inflammatory function, become more efficient at phagocytosis, and may be part of the mechanism whereby abx leads to a reduction in amyloid. Another piece of evidence which supports this hypothesis is that when colony stimulating factor 1 receptor (CSF1R) antagonists are given to abx-treated mice to deplete microglia, abx-mediated reduction in amyloid pathology does not occur [8]. This suggests that microglia are important for the abx-mediated reduction in amyloid pathology. Microglia are also strongly affected in germ-free AD mice compared to AD mice that are conventionally housed. Harach and colleagues [14] showed that GF APPPS1-21 mice had reduced ionized calcium binding adapter 1 (Iba1) + microglia in the brain at 3.5 and 8 months compared to conventionally housed mice [14]. In contrast, Mezo and colleagues [12] observed an increase in Iba1 + microglia in GF 5XFAD mice in the hippocampus at 4 months of age compared to 4-month-old conventionally housed mice. Microglial bulk RNAseq revealed an activated microglial signature in GF 5XFAD mice characterized by upregulation of *Apoe, Trem2, Axl, Cst7, Cd9, Itgax,* and *Clec7a* and downregulation of *P2ry12*. Lastly, GF 5XFAD mice had more efficient microglial phagocytosis of Aβ compared to conventionally raised mice [12]. Colombo and colleagues used GF APPPS1-21 mice to show that GMB-derived SCFAs control microglial transcriptomic state [13]. While GF APPPS1-21 have a decrease in SCFAs, plaque associated microglia, and Aβ plaques, administration of SCFAs in GF APPPS1-21 mice resulted in an increase in plaque associated microglia and Aβ plaques. Nanostring transcriptomic analysis revealed an activated microglial state, characterized by increased expression of several genes in the APOE-TREM2 pathway in SCFA treated GF APPPS1-21 mice. These results suggest that gut-derived SCFAs mediate microglial state that can modulate Aβ plaques in the brain [13]. While germ-free mice are useful models for studying GMB contribution to disease, they have a caveat in that they possess several developmental defects which may effect disease phenotype and may not be translationally relevant [33–35]. Peripheral [36] and central immune development [34], neurotransmission [37], and neurogenesis [38] may be altered by GF conditions and could confound studies using these models. It is useful to combine GF-based GMB manipulation with other less severe GMB manipulations to validate importance of findings to a disease model. In addition to microglia, astrocytes are the other main cell type in the brain that are involved in the innate immune response. The effect of GMB manipulation on reactive astrocyte modulation in AD has not been explored extensively. Recent evidence from our group suggests that GMB perturbations via abx and germ-free environment in the APPPS1-21 mouse model of amyloidosis reduces GFAP + reactive astrocytosis, astrocyte complement C3 expression, astrocyte recruitment to amyloid plaques, and alters astrocyte morphology in male mice. FMT from untreated APPPS1-21 mice into abx-treated APPPS1-21 mice restores astrocytic changes suggesting that the GMB is indeed regulating GFAP + astrocyte reactivity to amyloid plaques [39]. In the context of Multiple Sclerosis (MS), it has been shown that gut-derived metabolites, such as derivatives of tryptophan (indole, indoxyl-3-sulfate, indole-3-propionic-acid, indole-3-aldehyde) can directly modulate astrocyte reactivity [40]. Rothhammer et al. (2016) showed that gut-derived tryptophan metabolites can increase aryl hydrocarbon receptor (Ahr) signaling, which can suppress astrocytic inflammation in the EAE model of MS [40]. Additionally in MS, gut-derived metabolites can modulate gene expression in other CNS cells, such as microglia [41] or meningeal natural killer (NK) cells [42], which in turn, can regulate astrocyte reactivity. Another study by Rothhammer et al. (2018) showed that gut-derived tryptophan metabolites can modulate TGFɑ and VEGF-B signaling in microglia via Ahr. These signaling pathways then control astrocytic inflammatory state. Sanamarco et al. (2021) found that the GMB regulates the expression of IFN-γ in NK cells. NK-derived IFN-γ regulates the induction of LAMP1^+^ TRAIL^+^ astrocytes, which induce pro-inflammatory T-cell apoptosis. Furthermore, SCFAs in vitro can modulate astrocytic gene expression in a sex-dependent manner [43]. These collection of studies indicate that the GMB is capable of influencing astrocyte phenotype. Because astrocytes are phagocytic cells, have immune function, and can contribute to neurodegeneration [44], amyloid [45–48], and tau deposition and spread [49, 50], it is likely that GMB-controlled astrocytes are important in AD. Therefore, more work needs to be done to fully understand the importance of GMB-mediated control of astrocytosis in AD and whether the GMB-astrocyte axis could be therapeutically targeted.

#### GMB and Blood brain barrier/Peripheral immunity

BBB breakdown in neurodegenerative disease is likely important for GMB and peripheral involvement in disease progression. After BBB breakdown, peripheral immune cells, cytokines, and metabolites can more easily enter the brain and have an effect on brain resident cells and neuropathological progression [51]. While the connection between the GMB, the blood brain barrier, and AD has not been well established, there have been studies showing that the GMB can regulate BBB permeability through GMB-derived metabolites [52, 53]. One study showed that germ free mice have increased BBB permeability because of reduced expression of tight junction proteins during fetal life that persisted into adulthood. Recolonization of germ-free mice with conventional mouse flora reversed these effects. Additionally, SCFAs were able to modulate expression of tight junction proteins as well, suggesting that GMB alterations in SCFA production is the mechanism whereby BBB permeability is altered [52]. Another study showed that a different GMB-derived metabolite, methylamine trimethylamine N-oxide (TMAO), was able to enhance BBB integrity by altering expression of annexin A1, a tight junction protein. TMAO was also able to limit LPS-mediated memory impairment by limiting microglial and astrocyte-mediated neuroinflammation [53]. Together, these studies suggest that the GMB, through metabolites, can influence BBB integrity. BBB breakdown is well documented in AD, so it is possible that GMB dysbiosis can modulate this process and lead to more severe BBB breakdown. However, additional studies are needed to more fully understand the link between the GMB, BBB, and AD.

In terms of how GMB perturbation alters the peripheral immune system in AD models, Minter et al. reported increased plasma CCL11, IL1β, IL2, IL3, and stem cell factor (SCF) and reduced IL6 in APP/PS1 mice treated with abx compared to controls [6]. Furthermore, an increase in regulatory T cells (T-regs) in the blood and brain was observed in abx-treated mice compared with controls [6]. However, there were no significant differences in CD4 or CD8 T-cells [6]. T-regs are known to reduce inflammation [54], which would be consistent with the reduced neuroinflammation that has been seen across AD models in the context of abx or GF mice [5–8, 12–14]. Another study showed that cytokine differences in the blood of abx-treated APPPS1-21 mice are sex-specific [7]. Abx-treated male APPPS1-21 mice had increased plasma anti-inflammatory cytokines, such as insulin like growth factor binding protein 3, IL6, and IL10 and decreased pro-inflammatory cytokines, such as eotaxin1, IL-1β, IL2, IL3, IL17a, and CCL5 compared to controls. These peripheral changes were correlated with decreased Aβ plaque deposition and microglial activation exclusively in male mice in the brain [7]. However, female abx-treated mice had an increase in pro-inflammatory cytokines, including IL1β, IL5, IL9, and IL17a. Additionally, another study observed a decrease in Th1 + cells in the brain after abx treatment in 5XFAD mice. This was also associated with reduced microglial activation. Furthermore, FMT from 5XFAD mice into WT mice with hippocampal Aβ injection increased Th1 + cells in the brain while decreasing Th2 + cells [55]. Another study observed a reduction in IFN-γ, IL2, IL1β, and IL5 in the blood of GF APPPS1-21 mice compared to conventionally housed mice [14]. Other researchers showed that FMT restored an abx-mediated reduction in basic fibroblast growth factor (bFGF) and granulocyte–macrophage colony stimulating factor (GM-CSF) [8]. Overall, these studies suggest that there are plasma cytokine and peripheral immune cell changes in a GMB-perturbed state in amyloidosis models. However, there is variability between studies of which specific cytokines are altered. More sensitive and unbiased approaches are likely needed to resolve differences between studies. It should be noted that many of the cytokine studies discussed above are based on membrane-based cytokine arrays, which are only semi-quantitative [5–8], and future label-free proteomic/mass spectrometry approaches of fractioned plasma will be necessary to assess the entire repertoire of soluble factors that are modulated by the GMB.

#### Vagus nerve-mediated connection between gut and brain

While secretory products, such as gut-derived metabolites may mediate gut-brain connection, there is also a direct connection via the vagus nerve [56]. The vagus nerve is the longest cranial nerve in the body and stretches from the large intestine to the brain. It is critical in involuntary parasympathetic control of digestion, heart rate, respiration, and other vital functions. The vagus nerve allows for bidirectional communication between the gut and the brain and has 80% afferent and 20% efferent fibers. Thus, microbiota-derived molecules can affect vagus nerve firing and some of these molecules can travel to the brain via the vagus nerve [56]. Several taxonomic groups of bacteria can synthesize neurotransmitters, such as dopamine, serotonin, acetylcholine, and gamma-aminobutyric acid (GABA), which have neuroactive effector functions [56]. Several studies have shown that gut-derived neurotransmitters are important in mood regulation and psychiatric disease [57, 58]. Additionally, gut-derived hormones produced by enteroendocrine cells can bind to receptors on vagal afferent fibers [59]. Furthermore, SCFAs can directly also act on afferent vagal fibers [60]. It’s possible that vagus nerve stimulation can affect immune activity in the brain. Stimulation of the vagus nerve has been reported to decrease neuroinflammatory reaction to systemic lipopolysaccharide administration and induce anti-inflammatory microglial phenotype in an AD mouse model [61, 62]. Moreover, stimulation of the vagus nerve has also been shown to release acetylcholine, which has an anti-inflammatory effect on macrophage gene expression in the periphery [63]. It is possible that gut-derived signaling via the vagus nerve may be involved in AD progression; however, this has not yet been tested. If vagus nerve signaling is important to GMB involvement in AD, it may be through gut-derived neurotransmitter, metabolite, or hormonal mediated vagal stimulation altering immune responses in the periphery and brain and subsequent altered immune cell reaction to amyloid and tau pathology.

#### Anti-microbial properties of amyloid beta

While most AD research assumes Aβ is detrimental for the brain and has no beneficial physiological function, Robinson and Bishop initially introduced the hypothesis that Aβ may be produced to sequester toxic substances, like metal ions and pathogens, for presentation to phagocytes [64, 65]. All humans produce Aβ40 and Aβ42 throughout life and levels of these proteins are relatively high in both the CSF and serum throughout life [66, 67]. Moreover, Aβ plaques can be present in cognitively normal individuals [68]. This suggests that perhaps Aβ is present for a reason and serves a beneficial physiologic purpose in the human body. This purpose may be to potentially bind and sequester pathogenic bacteria and viruses as a first line innate immune defense. Several bacteria and viruses have been shown to enhance amyloidogenic processing and subsequent Aβ production [69–74]. Furthermore, Aβ plaques in humans have been shown to contain viral and bacterial DNA [73, 75]. Aβ has been shown to have similar structure to other antimicrobial peptides as well [76]. A prominent study supporting the idea that Aβ functions as an antimicrobial peptide is that by Kumar and Colleagues (2016) [77]. Kumar et al. showed that Aβ protects against infection in mouse, cell culture, and *C elegans* models [77]. This study suggests that Aβ oligomers bind to microbial cell walls via heparin binding domains, Aβ protofibrils prevent pathogen attachment to host cells, and Aβ fibrils trap pathogens and present them to phagocytes [77]. Given the evidence for Aβ mediated protection against infection, the idea that AD may have an infectious etiology is possible. Perhaps Aβ is initially increasingly produced as a response to real or perceived brain infection, but too much Aβ production eventually becomes detrimental by inducing neuronal toxicity, excessive neuroinflammation, and pathological tau seeding. Additionally, the question of whether there is a resident brain microbiome is under investigation [78]. Several sequencing studies have identified microbial sequences in the human brain parenchyma, but it is difficult to determine whether these sequences are indeed resident bacteria or due to contamination[79–82]. If a human brain microbiome does exist, aging may lead to pathogenic bacteria dominance in the brain, excessive Aβ production in response, and subsequent Aβ-induced toxicity.

### Microbiome-related therapeutic strategies for AD

#### Probiotics/prebiotics

**Fig. 2:**
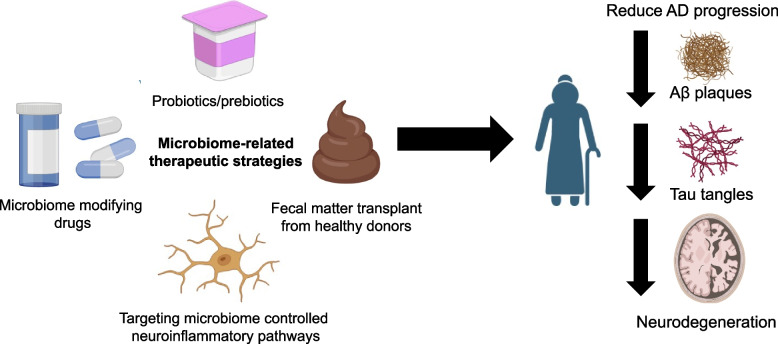
Microbiome-related therapeutic strategies for AD. Preliminary evidence from mouse and human studies suggests that probiotics/prebiotics, fecal matter transplant from healthy donors into AD patients, microbiome modifying drugs, and direct targeting of gut microbiome controlled neuroinflammatory pathways may potentially be disease modifying therapeutic strategies and may reduce amyloid, tau, and neurodegeneration

Because of the evidence suggesting the GMB plays a role in AD pathogenesis, therapeutic strategies targeting the GMB may be useful for modifying disease progression. One class of therapeutics that may be effective in modifying the GMB and altering disease progression is probiotics/prebiotics (Fig. 2) [83, 84]. Probiotics are comprised of formulations of live, beneficial bacteria. The most common bacteria in these formulations are *Lactobacillus* and *Bifidobacterium* [83]*.* Upon consumption, probiotics are designed to restore GMB dysbiosis and reduce disease progression and severity. Prebiotics are a type of substrate that the human body cannot digest, but are digested and used by bacteria and lead to a health benefit. Prebiotics are designed to be used as fuel for beneficial bacteria in the human gut [83]. There have been several studies showing the efficacy of probiotics/prebiotics in AD mouse models. However, there is variability depending on formulation, dose, treatment paradigm, and AD model used. Kaur et al. (2021) treated 6-month-old APP^NL−G−F^ for 8 weeks with probiotic formulation VSL#3 (*Lactobacillus plantarum, Lactobacillus delbrueckii subsp. Bulgaricus, Lactobacillus paracasei, Lactobacillus acidophilus, Bifidobacterium breve, Bifidobacterium longum, Bifidobacterium infantis, and Streptococcus salivarius subsp. Thermophilus*) [85]. These investigators found that VSL#3 altered the GMB composition, boosting levels of *Clostridia, Lachnospiracea* and *Akkermansia* and increased levels of acetate, lactate, butyrate, isobutyrate, and propionate in the serum. However, only lactate and acetate were increased in the brain. Although there were clear GMB and SCFA changes when APP^NL−G−F^ were given VSL#3, there were no changes in Aβ, glial fibrillary acidic protein (GFAP), Iba1, or the proliferative marker, Ki-67. This outcome could be because 6 months could be too late to initiate probiotic treatment in the APP^NL−G−F^ model in which pathology is already quite severe at that age [85, 86]. In contrast, Abdelhamid et al. (2022) showed that 3-month-old APP^NL−G−F^ mice treated with *Bifidobacterium breve* for 4 months had reduced Aβ, Iba1, and pro-inflammatory cytokines, as well as increased ADAM10 and synaptic proteins [87]. The earlier age of treatment could explain the difference in result compared to Kaur et al. [85, 87]. An earlier report from this same group showed a reduction in immune responses by bulk RNAseq in *Bifidobacterium breve-*treated mice injected with Aβ compared to vehicle control [88]. In another study, Asl et al. (2019) treated Aβ-injected rats with probiotics (*Lactobacillus acidophilus, Bifidobacterium bifidum and Bifidobacterium longum*) or vehicle [89]. These investigators reported that probiotic-treated rats performed better in Morris water maze testing compared to vehicle-injected rats. Furthermore, the vehicle-treated rats had suppressed LTP which was restored with probiotics [89]. A pair of papers from Bonfili et al. showed the beneficial effects of SLAB51 *(Streptococcus thermophilus, Bifidobacteria longum, Bifidobacteria breve, Bifidobacteria infantis, Lactobacilli acidophilus, Lactobacilli plantarum, Lactobacilli paracasei, Lactobacilli delbrueckii subsp. bulgaricus, Lactobacilli brevis)* treatment on 8-week-old 3xTg mice for 4 months [90–92]. SLAB51 treatment improved performance on the novel object recognition test, reduced brain damage, decreased Aβ plaques, increased SCFAs, and decreased plasma cytokine levels [90]. Another study by the same group showed that SLAB51 may have its protective effect by increasing Sirtuin-1, a protein deacetylase, which is able to protect cells from oxidative stress [91]. Probiotics may also be effective in combination with other efficacious treatments, such as exercise. Abraham et al. (2019) showed that a probiotic formulation (*Bifidobacterium longus, Lactobacillus acidophilus*, vitamin A, vitamin D, omega 3 fatty acids in cod liver oil, and vitamins B1, B3, B6, B9, B12) combined with exercise reduced Aβ and increased cognitive performance of APP/PS1 mice in the Morris water maze [93]. Cao et al. (2021) showed that 4-month-old APP/PS1 mice treated with *Bifidobacterium lactis* Probio-M8 for 45 days exhibited fewer Aβ plaques, had an alteration of their GMB composition, and increased cognitive performance in a Y-maze test [94].

In addition to probiotics, prebiotics have also been shown to be effective in amyloid models of AD. Liu et al. (2021) treated 5XFAD mice with prebiotic mannan oligosaccharide for 8 weeks from birth and found that it decreased cognitive deficits, Aβ plaques, reduced oxidative stress, microglial activation, and altered the GMB. They found that GMB-induced changes in the brain were likely mediated by SCFAs as supplementation with SCFAs produced the same effects [95]. Chen et al. (2020) treated 5XFAD mice with the prebiotic R13 that is a tropomyosin receptor kinase B (TrkB) agonist and found that the compound blocks the proinflammatory C/EBPB/AEP pathway in the gut and reduces amyloid-positive signals in the gut, as well [19].

In addition to studies in AD model mice testing probiotics, there have also been small trials testing probiotics in human AD patients. Akbari et al. (2016) (*N* = 60) conducted a randomized, double-blind clinical trial comparing outcomes in AD patients receiving probiotic milk (*Lactobacillus acidophilus, Lactobacillus casei, Bifidobacterium bifidum, and Lactobacillus fermentum*) or control milk (200 ml/day) for 12 weeks. They found that probiotic-treated patients had a significant improvement in their Mini Mental State Examination (MMSE) cognition score and a decrease in plasma malondialdehyde, a marker of oxidative stress, and a decrease in plasma C-reactive protein, a general marker of inflammation [96]. However, another trial from the same group (*N* = 60) in patients with severe AD showed that 12 weeks of probiotic treatment (*Lactobacillus fermentum, Lactobacillus plantarum, Bifidobacterium lactis, Lactobacillus acidophilus, Bifidobacterium bifidum, and Bifidobacterium longum)* was insufficient to alter cognitive score on the Test Your Memory test compared to controls. Additionally, the probiotics did not alter levels of inflammatory cytokines or markers of oxidative stress in plasma [97]. These differing results may be due to differing formulas of probiotics used or the severity of AD in patients included. This likely indicates that probiotics may be more clinically beneficial earlier in disease course when pathology is not as severe. In line with this speculation, Xiao et al. (2020) (*N* = 80) conducted a randomized, double-blind trial to test whether probiotics would be clinically beneficial in MCI patients. These investigators reported that 16 weeks of treatment with *Bifidobacterium breve A1* resulted in an improvement in Repeatable Battery for the Assessment of Neuropsychological Status (RBANS) and the JMCIS tests in MCI patients compared to placebo [98]*.* Probiotics may also be beneficial in combination with other treatments. Tamtaji et al. (2019) (*N* = 79) showed that probiotic (*Lactobacillus acidophilus, Bifidobacterium bifidum, and Bifidobacterium longum)* combined with selenium supplementation in AD patients resulted in synergistic improvements in MMSE score, reductions in CRP, reductions in total antioxidant capacity, lower insulin levels, lower LDL levels, and lower serum triglycerides [99]. A meta-analysis testing the available data on whether probiotics would be therapeutically beneficial for AD was conducted by Den et al. (2020). Through 5 studies with 297 patients, they found that overall probiotics resulted in an improvement of cognition, a reduction in plasma malondialdehyde, and a reduction in plasma CRP levels compared to controls [100]. Overall, these results suggest that probiotics could potentially be clinically useful for AD. However, more long-term, larger trials are needed to confirm their usefulness for AD as the trials described above are short-term with low sample size. Additionally, with the advent of effective blood-based biomarkers for AD [101], testing probiotics before the onset of clinical symptoms may be the most effective strategy to modify disease progression. Furthermore, although none of the studies discussed assess GMB changes in patients after probiotic treatment, efficacy in probiotic administration in altering clinical progression suggests that altering the GMB may be effective for modifying AD-related neuropathology and disease progression.

#### Fecal matter transplant

Another therapeutic strategy involving manipulation of the GMB is fecal matter transplant (FMT) (Fig. 2). FMT is currently used for *Clostridium difficile* infection relapses [102], but potentially could be therapeutic for a variety of conditions where the GMB has been linked to pathological progression [103, 104]. In AD, a pair of studies were published recently demonstrating the effectiveness of FMT in reducing pathology in AD mouse models [105, 106]. Sun et al. (2019) showed that administering FMT from WT mice into 6-month APP/PS1 mice for 4 weeks resulted in a reduction in Aβ, tau hyperphosphorylation, increased levels of synaptic proteins, and decreased cyclooxygenase-2 (cox-2) and CD11b + microglia [105]. Kim et al. (2019) corroborated these findings in the ADLP^APT^ mouse model of AD by showing that treating ADLP^APT^ mice from 2 months of age with WT FMT until 6 months of age resulted in a reduction in Aβ, tau phosphorylation, Iba1 + microglia, GFAP + astrocytes, Ly6C^high^ monocytes, and better performance in contextual fear conditioning and Y maze [106]. In contrast, Dodiya et al. (2022) found that FMT from WT mice into abx-treated APPPS1-21 mice from postnatal day 25 until 9 weeks of age resulted in an increase in amyloid and microglial activation [8]. While apparently contradictory to the studies by Kim et al. and Sun et al., the FMT studies by Dodiya et al. were performed in abx-treated animals with a perturbed GMB that exhibited reductions in Aβ amyloidosis and microglial activation and hence the FMT simply restored these parameters to those observed in APPPS1-21 mice without any GBM perturbations. With that in mind, more studies are needed across several amyloid and tau models and timepoints to determine whether FMT could be a viable therapeutic strategy for AD.

#### Other types of therapeutic modulation involving the GMB

Drugs that are able to modify the GMB could potentially be useful for AD (Fig. 2). Sodium oligomannate (GV-971) manufactured by GreenValley pharmaceuticals was reported by Wang et al. (2019) to reduce AD pathology in the 5xFAD mouse model through a mechanism involving GMB modification [55, 107]. The scientists reported that GV-971 therapeutically suppresses GMB dysbiosis, reduces peripheral inflammation and subsequent neuroinflammation. Furthermore, in a 36-week, multicenter, randomized phase 3 clinical trial in China (*N* = 818), GV-971 met its primary endpoint [108]. However, the study failed to meet its secondary endpoints, but the compound was still approved by the Chinese FDA. Global clinical trials with sites in North America and Europe have been initiated but currently on hold due to financial issues related to the COVID-19 pandemic. Further studies on this compound and other GMB modifying drugs be of great interest to determine whether GMB modification could be a useful therapeutic mechanism for targeting AD (Fig. 2).

An upcoming class of therapeutics with potential to target the GMB are engineered probiotics [109–111]. Engineered probiotics genetically manipulate bacterial species to produce beneficial metabolites/compounds in response to a particular stimulus [109–111] and have so far been tested in models of gastrointestinal disease, such as inflammatory bowel disease [112] and *Clostridium difficile* infection [113]. Additionally, bacteria have been engineered to release anti-cancer therapies [114–116]. This class of therapeutics likely would also be beneficial in brain diseases in which the GMB is involved, such as AD, PD, and MS.

Another important therapeutic strategy that is GMB-related is harnessing bulk and single-cell RNAseq to identify brain-wide and cell-type specific pathways that are regulated by the GMB and target those pathways/cell types (Fig. 2). This might be a more effective method to direct GMB manipulation as a therapeutic strategy since there are considerable variations in how particular GMB manipulations affect individuals. However, pathways regulated by the GMB may be more ubiquitous. For example, Sanmarco et al. (2021) recently identified a GMB-regulated anti-inflammatory TRAIL + /LAMP1 + astrocyte subtype [42]. Induction of this astrocyte subtype could be therapeutic for several neurological diseases. Similar therapeutic strategies could be applied to GMB-regulated microglial, neuronal, and oligodendrocyte subtypes/substates. Additionally, manipulation of GMB-regulated peripheral inflammatory subtypes/substates could be beneficial as well. This type of strategy could be done by using adeno-associated vector virus therapy [117] with a cell-type specific promoter or by using ligand-conjugated antisense oligonucleotides [118]. Importantly, for implementation of this strategy there likely needs to be better means of cell-type specific therapeutic modulation. Furthermore, modulation of pathways identified in bulk RNAseq may be important therapeutically as well. Chen et al. (2020) identified that the C/EBPβ/AEP inflammatory pathway in the brain is regulated by the GMB and may influence amyloid pathology [19]. Targeting this pathway could potentially be beneficial for AD.

### Potential links between the GMB and AD that need further investigation

The effort to understand the role of the GMB in AD is relatively new with the first studies in humans suggesting that the GMB is altered in AD patients compared to healthy controls and mouse studies suggesting GMB manipulations alter AD pathology published in 2016 and 2017. As such, there are several potential mediators of the relationship between the GMB and AD pathology that have not yet been explored. In this section, we describe the potential connections which may exist between the GMB and diet, sleep, and exercise and the development and progression of AD.

#### Diet

The connection between diet and AD is well established. Epidemiologic evidence indicates that high-fat diets and obesity are associated with increased risk for developing AD and dementia [119–123]. Furthermore, the incidence of AD is higher in countries that typically consume high-fat diets as opposed to low-fat diets. Several mouse studies have shown that AD model mice that consume high-fat diets have accelerated neuropathology. Increased Aβ plaques in the brain after consumption of a high fat diet has been observed in the APP/PS1 [124, 125], 5XFAD [126], APP23 [127], and APP^NL−F^[128] models of amyloidosis. Additionally, several studies have shown that a high fat diet can result in an increase in neuroinflammation [124, 125, 127, 128] and decreased performance on AD-related behavior tests [125, 127–132]. However, there have also been some studies that have not observed an effect of high fat diet on AD-related neuropathology [129, 132, 133]. Differences in outcomes in mouse models may be due to model, sex, and treatment differences. However, human data suggests, in general, that high-fat diets and obesity are risk factors for AD. Conversely, there is evidence suggesting that the Mediterranean diet, which is filled with plant-based foods and healthy fats, may protect against AD [134–140]. Ballarini et al. (2021) recently observed that higher Mediterranean diet adherence led to higher grey matter volume, better memory, lower Aβ, and lower phosphorylated tau [141]. Additionally, the ketogenic diet, which involves high fat and low carbohydrate consumption, has also been shown to potentially have benefits in AD [142–145]. Studies in mouse models of AD have shown that the ketogenic diet can improve memory, reduce amyloid plaques, reduce neurodegeneration, and neuroinflammation [146–148]. Additionally, the ketogenic diet has been shown to alter GMB composition and neurovascular function, which could be beneficial for AD [149]. Interestingly, Nagpal et al. (2019) found that a modified Mediterranean-ketogenic diet could alter the GMB and SCFA production in human MCI which correlated with amyloid measured in cerebrospinal fluid (CSF) [150]. Overall, the evidence seems clear that diet can influence AD risk and progression.

Diet is one of the most important factors that influences GMB composition. For instance, Mediterranean, ketogenic, vegan, and gluten-free diets alter GMB composition in humans [151–157]. Furthermore, the GMB plays a large role in the development and progression of obesity and metabolic syndrome [158, 159]. GMB composition is altered in obese individuals and in mice [160, 161]. Furthermore, GF mice have reduced body fat compared to conventionally raised mice even though they require a much higher energy intake to maintain the same weight as conventionally raised animals [162]. Additionally, GF mice that consumed a high fat, high carbohydrate diet gained less weight than conventionally raised mice [163]. Another study showed that colonization of GF mice with cecal content from lean or obese donors resulted in the obese recipient mice having a much larger percentage of body fat compared to mice colonized with cecal content from lean donors [164]. Taken together, these studies indicate that the GMB is heavily involved in the development and progression of obesity and energy homeostasis. The GMB may regulate peripheral inflammation, which modulates the development and progression of obesity [165–168].

There is convincing evidence of the connection between diet, GMB, and AD [169, 170]. The connections between diet and AD, diet and GMB, and GMB and AD have been studied. However, there is a lack of published experiments where all 3 factors are studied together. Nagpal et al. (2019) conducted a study using 17 patients (11 with MCI, 6 cognitively normal) where the patients adhered to a modified Mediterranean-ketogenic diet for 6 weeks followed by 6 weeks of adhering to a diet recommended by the American Heart Association. GMB, plasma SCFAs, and plasma AD markers were measured before and after the diets. These investigations revealed that each diet modified the GMB and SCFA production in distinct ways and some of these changes correlated with CSF Aβ-42 [150]. Future longitudinal studies need to be conducted to determine whether diet interventions may modify AD phenotypes through the GMB. Additionally, more animal model experimentation is needed to determine whether GMB changes are necessary for diet interventions to modify AD phenotypes. There is a likely connection between these components in that diet modifies the GMB, which regulates peripheral and neuroinflammation, which can in turn affect the progression of AD pathology.

#### Sleep

Sleep disturbances and circadian rhythm dysfunction are heavily implicated in AD [171]. AD patients often have disrupted sleep wake cycle and are often increasingly awake at night and sleepy during the day [172]. They also spend less time in slow wave [173, 174] and rapid eye movement (REM) sleep [175, 176], both of which are critical for memory consolidation and cognition [177, 178]. Furthermore, poor sleep and sleep fragmentation may predict AD and subsequent dementia [179–181]. Additionally, cognitively normal adults who self-report sleep issues are more likely to have amyloid pathology in their brains on PET scan [182]. Aβ levels have been shown to be regulated by the sleep–wake cycle. When brain interstitial fluid (ISF) is sampled in mice using microdialysis, levels of Aβ are increased when mice are awake and decrease when the mice are asleep [183]. This finding was also confirmed in human AD patients [184]. These diurnal oscillations in Aβ levels in brain ISF are dissipated and the sleep–wake cycle is disrupted after Aβ plaque formation in the APP/PS1 mouse model of amyloidosis. However, amelioration of Aβ plaque pathology with Aβ immunotherapy restored a normal sleep wake cycle and diurnal oscillations in Aβ levels in the mice [185]. This study directly implicates Aβ plaque pathology in sleep–wake cycle and diurnal Aβ oscillations. The diurnal Aβ oscillations are also thought to be a result of differences in neuronal activity between sleep and wakefulness. During sleep, neuronal activity is reduced and during wakefulness, it is increased. Lower neuronal activity during sleep likely leads to less Aβ production [186–188]. Sleep deprivation exacerbates AD pathology [183, 189, 190], which clearly connects poor sleep quality with progression of AD. Additionally, circadian rhythm dysfunction is implicated in AD [191]. Mouse models of AD show circadian dysfunction over time [191]. Tranah et al. (2011) found that circadian dysfunction could predict future development of AD [192]. Furthermore, single nucleotide polymorphisms in the Clock gene are associated with AD [193–195].

There are several lines of evidence that suggest the GMB may influence sleep quality and that sleep quality may influence GMB composition. Antibiotic-mediated perturbation of the GMB can result in fragmented NREM sleep [196]. On the other hand, sleep disruption can lead to changes in GMB composition. Voigt et al. (2016) found that mutant Clock mice that have disrupted sleep had a significantly altered GMB composition and lower taxonomic diversity compared to controls [197]. This effect was exacerbated by alcohol consumption, suggesting that poor sleep combined with other factors may alter the GMB even more compared to poor sleep alone. This may suggest a two-hit hypothesis whereby poor sleep can have a large effect on the GMB and predispose to various pathological conditions. Poroyko et al. (2016) observed a change in GMB composition after sleep fragmentation was induced in wildtype mice. As a result, these mice had adipose tissue inflammation and decreased insulin sensitivity. Furthermore, colonization from sleep-fragmented mice into germ-free animals caused these same phenotypes, implying that the GMB was mediating these effects [198]. Similarly, chronic sleep deprivation in 7-day old rats, resulted in GMB composition changes [199]. The mechanism whereby the GMB may influence sleep could be through microbial metabolites. Administration of butyrate has been shown to promote NREM sleep in rats and mice [200]. Additionally, a higher percentage of propionate in relation to total SCFA composition was associated with longer uninterrupted human infant sleep [201]. Sleep can also influence inflammation. Generally, sleep loss increases inflammatory responses. The connection between sleep and inflammation may be mediated by the GMB [202]. In addition to the connection of disrupted physiological sleep being associated with GMB changes, pathological sleep conditions are also associated with GMB alterations. Intermittent hypoxia associated with obstructive sleep apnea (OSA) is associated with GMB composition and diversity changes [203, 204]. Furthermore, OSA patients have altered GMB composition compared to HCs [205]. Similarly, insomnia and narcoleptic patients both have altered GMB composition compared to HCs [206, 207]. FMT from healthy donors improved sleep in irritable bowel syndrome patients [208]. Lastly, several probiotic/prebiotic formulations improve sleep [209–213], implying that increasing beneficial bacteria in the GMB can influence sleep outcomes.

The evidence suggests there is likely a connection between the GMB, sleep, and AD [214]. Although the connections between the GMB and sleep and sleep and AD have been studied, the connection between GMB, sleep, and AD together has not been extensively studied. This relationship is likely complex and bidirectional. Disrupted sleep may lead to gut dysbiosis, which can modulate AD pathology. It is also possible that gut dysbiosis may lead to sleep disruptions, which can then modulate AD pathology. There is likely a synergistic effect of these two scenarios, which contribute to AD pathogenesis. It is imperative that this connection is studied to better understand the mechanisms of AD progression and for therapeutic targeting of the sleep-GMB connection for AD.

#### Exercise

Human studies have shown that exercise is protective against age-induced cognitive decline and AD-related dementia risk [215–219]. This effect is likely mediated by enhanced adult hippocampal neurogenesis, brain derived neurotrophic factor signaling, and synaptic function as well as reduced neuroinflammation [220–222]. It is plausible that the GMB may mediate exercise’s beneficial effects on cognition. Exercise modifies the composition and diversity of the GMB in humans and in mice [223–226]. Masumoto et al. (2008) [223] was the first to report that 5 weeks of exercise training in mice resulted in GMB composition changes and an increase in cecal butyrate, which has since been recapitulated by other groups. Allen et al. (2017) [226] corroborated this finding in humans in a longitudinal study, where sedentary lean and obese female subjects participated in 6 weeks of supervised endurance-based aerobic exercise training 3 times/week and then went back to their sedentary lifestyle for 6 weeks. Allen et al. (2017) [226] showed that exercise induced shifts in GMB composition, and these changes were dependent on obesity status. Exercise increased fecal SCFA concentrations in lean but not obese participants. These effects were reversed after 6 weeks of ceasing exercise training suggesting that sustainment of exercise is necessary for long-term exercise-induced GMB alterations. Because exercise is protective in AD and exercise alters GMB composition, it is possible that the protective effect of exercise on AD progression is mediated by the GMB. Furthermore, the GMB can regulate neurogenesis and neuroinflammation, which are major mechanisms of exercise-induced benefits for cognition [35, 227]. To elucidate the connection between exercise, the GMB, and AD, future studies will need to take place in which AD model mice with GMB perturbations are given exercise training and AD-related pathophysiology is assessed compared to exercised AD mice without GMB perturbations. If the GMB is found to be an important mediator of exercise-induced benefits for AD, therapeutic strategies recapitulating an exercise-induced GMB state may be useful for AD.

## Conclusion

The GMB is a master regulator of inflammation in the body, and therefore is very important for the development and progression of diseases involving peripheral and central inflammation [228]. Understanding the mechanisms whereby the GMB can influence AD progression may reveal an important therapeutic target that could control several pathogenic mechanisms. Since initial studies revealed the profound effect of GMB alteration in AD-related pathology [5, 6, 14] and that AD patients have a significantly altered GMB composition compared to healthy controls [10, 11], there has been an explosion of interest in this subfield of AD. Based on studies to date, we now have a reasonable hypothesis that the GMB regulates peripheral inflammation and central inflammation likely through microbial metabolites (Fig. 1), which have an effect on AD pathology in the brain (Fig. 1). Administration of abx [5–8, 24] or germ-free conditions [12–14] in mouse models of amyloidosis results in reduced amyloidosis and microglial activation. Abx also increases the amount of anti-inflammatory regulatory T-cells in the blood and brain [6]. Peripheral immune changes likely influence central immune responses in microglia and other brain cells, which can affect amyloidosis and neurodegeneration (Fig. 1). Additionally, SCFAs produced by the bacteria in GMB can modulate amyloidosis [13]. Although contribution from several studies has given rise to a general hypothesis of how the GMB could modulate AD-related pathology, few specific targetable pathways have been identified. The rise of genomic technologies and bioinformatic tools will likely aid in the effort to find brain-wide and cell-type specific pathways that are influenced by the GMB and lead to modulation of AD pathology. Additionally, better models of human-relevant GMB manipulation are needed to better understand how mouse model experiments relate to the human disease. Collection of fecal matter from AD/MCI patients and healthy controls would be extremely valuable for mouse modeling. Colonization of germ-free mice with AD/MCI patient fecal microbiomes could help researchers better model human GMB changes in AD. Additionally, extra information, such as brain scan/blood-based biomarker data in combination with this would also benefit GMB/AD studies. We recommend that all AD clinical trials collect and store fecal samples from trial participants for use in GMB studies. Additionally, it will be of interest to understand how modifiers of the GMB, such as diet, geography, sex, aging, exercise, and sleep, may hinder efforts to therapeutically target the GMB. An important question to answer for translation is whether GMB-based therapies for AD can be generalized or whether they need to be personalized to the patient. To adequately answer this question there needs to be long-term human prevention and treatment trials using GMB-based therapeutic approaches. In summary, although research elucidating the connection between the GMB and AD has come far over a short period of time, using new tools and approaches will accelerate study to eventually fully understand and therapeutically target this connection.

## Data Availability

This study does not contain any data.

## Abbreviations

Aβ: Amyloid beta
AD: Alzheimer’s disease
ABX: Antibiotics
APP: Amyloid precursor protein
Beta-secretase 1: Bace-1
CSF1R: Colony-stimulating factor 1 receptor
DAM: Disease-associated microglia
FMT: Fecal matter transplant
GF: Germ-free
GMB: Gut microbiome
GFAP: Glial fibrillary acidic protein
HC: Healthy control
IBA1: Ionized calcium binding adapter 1
IHC: Immunohistochemistry
ISF: Interstitial fluid
MMSE: Mini mental state examination
NK cells: Natural killer cells
OPCs: Oligodendrocyte progenitor cells
SCFA: Short chain fatty acids
